# Head circumference as an epigenetic risk factor for maternal nutrition

**DOI:** 10.3389/fnut.2022.867727

**Published:** 2022-07-18

**Authors:** Maria J. Miele, Renato T. Souza, Iracema M. Calderon, Francisco E. Feitosa, Debora F. Leite, Edilberto A. Rocha Filho, Janete Vettorazzi, Jussara Mayrink, Karayna G. Fernandes, Matias C. Vieira, Rodolfo C. Pacagnella, Jose G. Cecatti, Preterm SAMBA study group

**Affiliations:** ^1^Department of Obstetrics and Gynaecology, University of Campinas (UNICAMP) School of Medicine, Campinas, SP, Brazil; ^2^Department of Gynaecology and Obstetrics, Botucatu Medical School, São Paulo State University (Unesp), Botucatu, SP, Brazil; ^3^MEAC–Maternity School of the Federal University of Ceara, Fortaleza, CE, Brazil; ^4^Department of Gynaecology and Obstetrics, Federal University of Pernambuco, Recife, PE, Brazil; ^5^Department of Obstetrics and Gynaecology, Maternity Hospital, Federal University of Rio Grande do Sul, Porto Alegre, RS, Brazil; ^6^Department of Obstetrics and Gynaecology, Jundiaí School of Medicine, Jundiaí, SP, Brazil; ^7^Division of Women and Children's Health, School of Life Course Sciences, Faculty of Life Sciences and Medicine, Kings College London, London, United Kingdom

**Keywords:** anthropometry, pregnancy, maternal nutrition, socioeconomic factors, newborn

## Abstract

Nutrition indicators for malnutrition can be screened by many signs such as stunting, underweight or obesity, muscle wasting, and low caloric and nutrients intake. Those deficiencies are also associated with low socioeconomic status. Anthropometry can assess nutritional status by maternal weight measurements during pregnancy. However, most studies have focused primarily on identifying changes in weight or Body Mass Index (BMI), and their effects on neonatal measures at present time. Whereas head circumference (HC) has been associated with nutrition in the past. When the mother was exposed to poor nutrition and unfavorable social conditions during fetal life, it was hypothesized that the intergenerational cycle was potentially mediated by epigenetic mechanisms. To investigate this theory, maternal head circumference (MHC) was associated with neonatal head circumference (NHC) in pregnant women without preexisting chronic conditions, differentiated by sociodemographic characteristics. A multiple linear regression model showed that each 1 cm-increase in MHC correlated with a 0.11 cm increase in NHC (β95% CI 0.07 to 0.15). Notwithstanding, associations between maternal and neonatal anthropometrics according to gestational age at birth have been extensively explained. Path analysis showed the influence of social status and the latent variable was socioeconomic status. A model of maternal height and head circumference was tested with effects on neonatal HC. The social variable lacked significance to predict neonatal HC in the total sample (*p* = 0.212) and in the South/Southeast (*p* = 0.095), in contrast to the Northeast (*p* = 0.047). This study highlights the potential intergenerational influence of maternal nutrition on HC, suggesting that maternal nutrition may be more relevant in families with major social vulnerability.

## Introduction

Famine and malnutrition at any stage of pregnancy can impose negative consequences for maternal and fetal health, that may perpetuate an inherited susceptibility to disease throughout the lifespan of an offspring (1). Nutrition science has been curiously investigating the associations between maternal nutrition thru dietary patterns population and effects on offspring for years (2). Nevertheless, the pregnancy is a short period of time to determine epigenetic effects at birth, considering there are strong links between health before pregnancy and results over generations (3) In contrast, socioenvironmental and genetic factors show a higher power of explanation in neonatal anthropometric measurements, such as neonatal head circumference (NHC) (4) Pre-pregnancy BMI is often used t o assess maternal nutritional status (5). However, maternal nutrition status is usually investigated by measuring clinical parameters when deleterious effects have already occurred and persist thru chronic undernutrition and adverse environmental exposures (6). HC is a measurement that depicts brain development in children (7) and it is also considered to be an indicator of nutritional status before birth (8). Offspring size is influenced by maternal and paternal genetics, and the intrauterine environment (9).

## Material and methods

This is an analysis of secondary objectives of a multicenter cohort study entitled “Preterm SAMBA–Screening and Metabolomics in Brazil and Auckland (10).” Singleton nulliparous pregnant women were included from 2015 to 2018 in five referral obstetric public hospitals, located in three geographical regions with diverse sociodemographic characteristics that best represented the diversity of social/ethnic aspects and eating habits in the Northeast in contrast to the South and Southeast of Brazil [Maternity Hospital of the State University of Campinas (CAISM) and Maternity Hospital from Botucatu Medical School in the Southeast; Maternity of the Hospital of Clinics, Federal University of Rio Grande do Sul in the South; and Clinics Hospital, Federal University of Pernambuco and Maternity School Assis Chateaubriand of the Federal University of Ceará in the Northeast].

Nulliparous pregnant women at 19 to 21 weeks of gestation and without pre-existing chronic conditions were invited to participate in this study. All women had an ultrasound scan performed before 20 weeks for confirmation of gestational age. Details of study methods and procedures are available in a previous publication (10).

Women with a history of three or more abortions, cervical alterations, major fetal anomaly, Mullerian anomalies, history of cervical cold conization, chronic corticosteroid use and pre-existing or self-reported disease, including hypertensive disease, previous diagnosis of diabetes mellitus, kidney disease, systemic lupus erythematosus or antiphospholipid syndrome, sickle-cell anemia and HIV-positive serology were excluded from the study. Women taking medications or supplements that could interfere in outcome evaluation were also excluded (Long term Steroids, Low-dose Aspirin 60–150 mg/24 h, Heparin/LMW Heparin, Calcium >1 g/24 h, Eicosopentanoic acid (Fish Oil), Vit C ≥1,000 mg and Vit E ≥ 400UI).

Maternal height was measured using a stadiometer. In all participating hospitals, the research team was previously trained to assess the first anthropometric measurements of the woman (weight, height, head circumference and arm circumference) at study entry (19–21 w) and repeated (weight, height and arm circumference) during follow-up visits (27–29 and 37–39 weeks). Neonatal head circumference was measured within the first 24 h of birth, by wrapping a tape around the head at the widest possible circumference of the head and recorded in centimeters. A flexible and non-stretchable tape was used to assess the measures following standardized criteria defined by the Food and Nutritional Surveillance System of the Ministry of Health (11). Newborns classified as having microcephaly or macrocephaly were excluded according to references of Ministry of Health of Brazil and the World Health Organization, below the 10th and above the 90th percentile according to sex and gestational age at birth (12, 13).Due to pragmatic reasons no assessment of intra or inter variability of maternal and neonatal anthropometric measurements was performed. The full planned study involved a lot of standardized procedures and processes and we needed to simplify the activity of the health professionals who received a specific training before starting data collection. Sociodemographic data were self-reported. Information collected was based on the Multiple Privacy Index which includes: income, occupation, relationship, number of people living in the household, schooling. The information collected was based on the Multiple Privacy Index (14) and Synthesis of Social Indicators of Brazil (15), such as income, occupation, kinship, number of people living in the household, education. All collected data were inserted into an electronic platform for data collection and storage (MedSciNet® AB, Sweden).

### Institutional review board statement

All women signed an individual two-way informed consent form before study admission. The Preterm-SAMBA study was conducted, in compliance with the Declaration of Helsinki (2013), following national and international regulations according to the Brazilian Resolution CNS 466/12. It was approved by the Institutional Review Boards of all participating centers (coordinating center protocol 20182318.8.0000.5404), in addition to the National Ethics Committee for Research (CONEP). All women included in this study signed an individual informed consent term, before admission. This manuscript follows the guidelines of the Strengthening the Reporting of Observational Studies in Epidemiology (STROBE) (14).

### Statistical analysis

Numerical variables are expressed as means and standard deviations. Categorical variables are represented by numbers and percentages. To assess data normality, a histogram was built, followed by the Shapiro-Wilk test. In descriptive statistics, to calculate the difference between selected variables, the Chi Square test or Student's *t*-test was applied according to categorical or numerical characteristics.

To manage data with variables of interest to fulfill study objectives, we selected neonatal HC measurements in response to maternal HC values. The assumption of residual independence was verified according to Durbin Watson, followed by the Breusch-Pagan test to assess homogeneity of error variance for the assumption of homoscedasticity. It was assumed that values higher than 0.05 did not violate this presumption. To eliminate the suggestion that a correlation existed between variables and multicollinearity implied, the Variance Inflation Factor (VIF) was used for assessment.

Multiple regression analysis was conducted to explore how maternal head circumference contributed to the prediction of neonatal HC measures. After evaluating the results, gestational age at birth was transformed into logarithm to control for heteroskedasticity effects of each conditional value, adequacy of regression analyses and inclusion in the adjusted model. The associations between estimates are shown by using Confidence Intervals for beta, as well as adjusted and non-adjusted R^2^. Interactions related to socioeconomic and demographic factors were also tested, including a confounding factor matrix for analysis of algorithm performance. Structural Equation Modeling (SEM) works with covariance patterns between features of interest and transforms the observed correlations into a system of equations that can mathematically describe one or more hypotheses related to causal relationships. This method is called path analysis. Factor analysis associated with path analysis can provide testing and describe causal relationships (16). Path analysis was conducted taking into account the influence of independent variables (maternal HC, Age, Schooling, and Income) on the dependent variable (neonatal HC). The original data was initially standardized by transforming measurements in a homogeneous scale using z-scores for analysis. The resulting model was tested using a confirmatory factor analysis (CFA) for validation of the proposed model. Plausibility indexes were considered acceptable parameter settings: Comparative Fit Index (CFI) = 0.90, Tucker-Lewis Index (TLI) = 0.95, Root Mean Square Error of Approximation (RMSEA) = 0.06, and Standardized Root Mean Square Residual (SRMR) = 0.08 (17). Since parameters of interest (path coefficients) and correlations are generally sensitive to extreme values, data selection was conducted to avoid possible errors, excluding births that occurred before the end of 37 weeks of pregnancy (18). The cutoff point of 37 weeks was chosen to align the descriptive and comparative objectives, without the influence of different pathological conditions or any truncated distribution.

A latent variable was developed using socioeconomic variables to define socioeconomic status. The model was structured with socioeconomic status as an effect indicator of the latent variable. Estimation of the latent variable named “Socio” was made by analysis of the variance and covariance of the following indicators: Age, Schooling and Income. Age was chosen rather than color due to the association between age and maternal anthropometric measurements and socioeconomic factors, in addition to a high correlation between country regions. A latent variable measurement model with effect indicators is the set of relationships (modeled as equations) where the latent variable is established as a predictor of the indicator. A bilateral *p*-value < 0.05 was considered statistically significant. Linear regression analyses were conducted using “Pac-Man” library package and “sjPlot,” while Path Analysis used “Lavaan” and “Sem” packages of R Core Team software (19).

## Results

**Figure 1:**
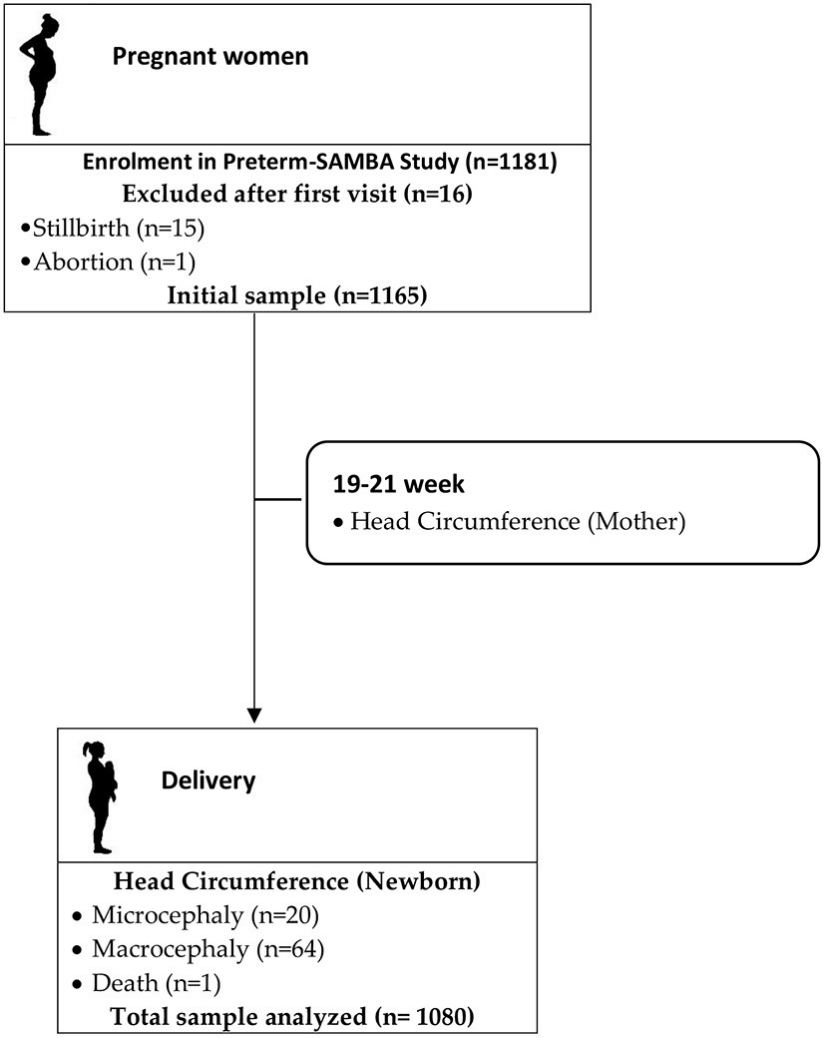
Flowchart of study population.

Initial data were obtained from 1,165 women at 19 to 21 weeks of gestation who gave birth at 23 to 42 weeks of gestational age. Eighty-five newborns classified as having microcephaly (n = 20) or macrocephaly (n = 64) and 1 death and 118 born before completing 37 weeks of gestation were excluded, resulting in a final sample of 962 mothers and their offspring (Figure 1).

Table 1 compared the profiles of Northeastern women with those from Southern/Southeastern women which indicates inequality among those regions.

**Table 1:** Distribution of anthropometric and sociodemographic characteristics according to the regions of Brazil (n = 962).

	**South/Southeast**	**Northeast**	***p*** **-value**
	**(*****n*** = **493)**	**(*****n*** = **469)**	
^a^>Maternal HC (cm)	55.27 ± 2.03	54.75 ± 1.83	<0.001
^b^Maternal Height (cm)	162.20 ± 6.85	159.15 ± 6.44	<0.001
^b^Maternal BMI (kg/m^**2**^)			0.769
Obese	86 (17.4)	73 (15.6)	
Overweight	121 (24.5)	122 (26.1)	
Adequate	201 (40.8)	185 (39.5)	
Underweight	85 (17.2)	88 (18.8)	
Maternal age (years)			<0.001
<20	108 (21.9)	148 (31.6)	
20–34	346 (70.2)	304 (64.8)	
>34	39 (7.9)	17 (3.6)	
Schooling (years)			<0.001
<12	310 (62.9)	350 (74.6)	
≥12	183 (37.1)	119 (25.4)	
Occupation			<0.001
Paid work	293 (59.4)	176 (37.5)	
Housewife	77 (15.6)	96 (20.5)	
Not working	123 (24.9)	197 (42.0)	
Maternal skin color/ethnicity			<0.001
White	183 (37.1)	119 (25.4)	
Non-white	310 (62.9)	350 (74.6)	
Family income (U$ per year)			<0.001
<3,000 (U$)	6 (1.2)	40 (8.5)	
3,000–6,000 (U$)	44 (8.9)	170 (36.2)	
>6,000–12,000 (U$)	140 (28.4)	171 (36.5)	
>12,000 (U$)	303 (61.5)	88 (18.8)	
Newborn outcomes			
^c^Newborn HC (cm)	34.22 ± 1.18	34.377 ±1.23	0.059
^d^Birthweight (kg)	3,220.99 ± 384.51	3,257.63 ± 434.91	0.166
^e^Length	48.55 ± 2.22	48.84 ± 2.19	0.042

*Numerical values expressed in means (±SD) categorical values expressed in %. p-values were obtained by Chi-square or t-test*.

*Missing data*:

(a) *Northeast = 25;*

(b) *Northeast = 1;*

(c) *South/Southeast = 38, Northeast = 92;*

(d) *South/Southeast = 1;*

(e) *South/Southeast = 7, Northeast = 22*.

*Values in bold mean they are significant*.

**Figure 2:**
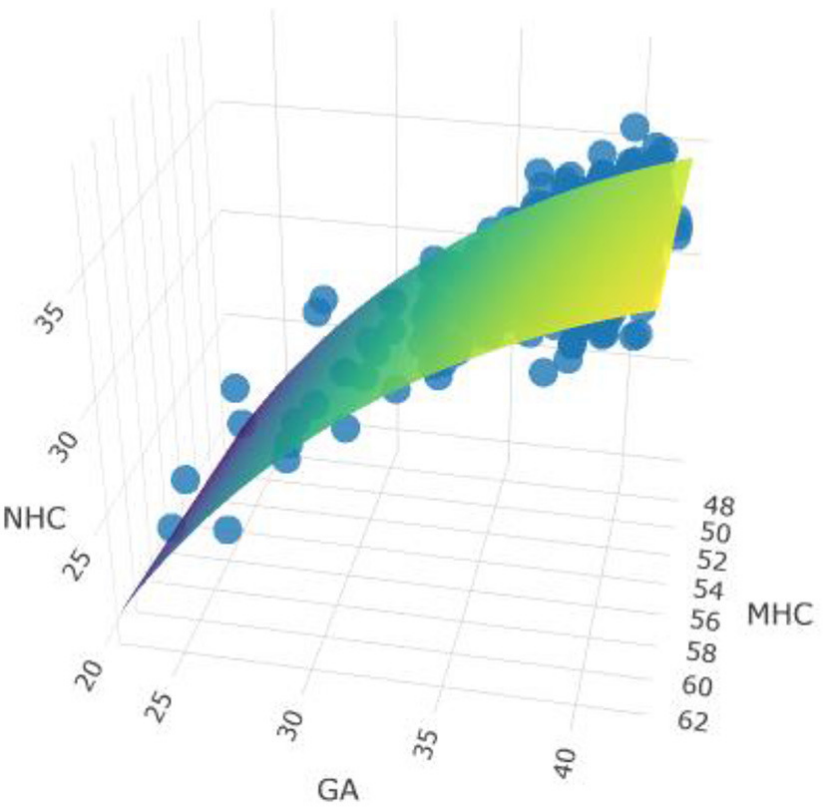
Model adjusted for gestational age and MHC and NHC measurements. Link to see model: https://rpubs.com/MariaMiele/899285. Observations 812, F-statistic, F statistics *p*-value <0.001. Adjusted R^2^/R^2^ajusted: 0.145/0.143.

Figure 2 shows the dynamic 3D-model adjusted for gestational age at birth for the association between head circumference measurements of pregnant women and their offspring at birth. It depicts the result of beta estimates for the association between MHC and NHC. Each increase in MHC of 1 cm was associated with an increase in NHC of 0.11 cm (β95%CI = 0.07–0.15), and gestational age (log) β = 13.78 (95%CI = 11.14–16.42). These results indicate that explanatory variables are related to NHC which is strongly relevant to *p*-value and significant for F statistics (*p* < 0.001).


$$\hat{\text{y}}\;=\beta_{0}+\;\beta_{1}({\text{x}}_{1})+\beta_{2}\log({\text{x}}_{2})$$

We repeated the process of multiple regression analysis using the stepwise AIC method, searching for new explanatory factors per region. In the Northeast, schooling was a significant factor. In contrast, the S/SE region showed that maternal age was a new explanatory factor. However, the power of explanation of maternal age was not as significant as adjusted R-squared for the model (Table 2).

**Table 2:** Multiple linear regression analysis of explanatory factors for the association between regions.

**Characteristics**	**Beta**	**95%CI**	***p*** **-value**
Northeast			
*Intercept*	−29.49	−43.89–−15.09	<0.001
MHC (cm)	0.17	0.10–0.23	<0.001
Gestational age (log)	14.85	11.03–18.68	<0.001
Schooling			
<12 years	−0.31	−0.57–−0.05	<0.001
≥12 years	–	–	–
South/Southeast			
*Intercept*	−16.93	−30.30–−3.56	0.013
MHC (cm)	0.07	0.02–0.12	0.007
Gestational Age (log)	12.92	9.32–16.53	<0.001
Maternal age			
≤ 19 years	−0.30	−0.55–−0.05	0.020
20–34 years	–	–	–
≥35 years	0.33	−0.05–0.71	0.088

*Northeast: Observations 357. F-statistic, p-value <0.001. R^2^/R^2^ajusted 0.211/0.204. South/Southeast: Observations 455. F-statistic, p-value <0.001. R^2^/R^2^adjusted: 0.133/0.125*.

*Values in bold mean they are significant*.

**Figure 3:**
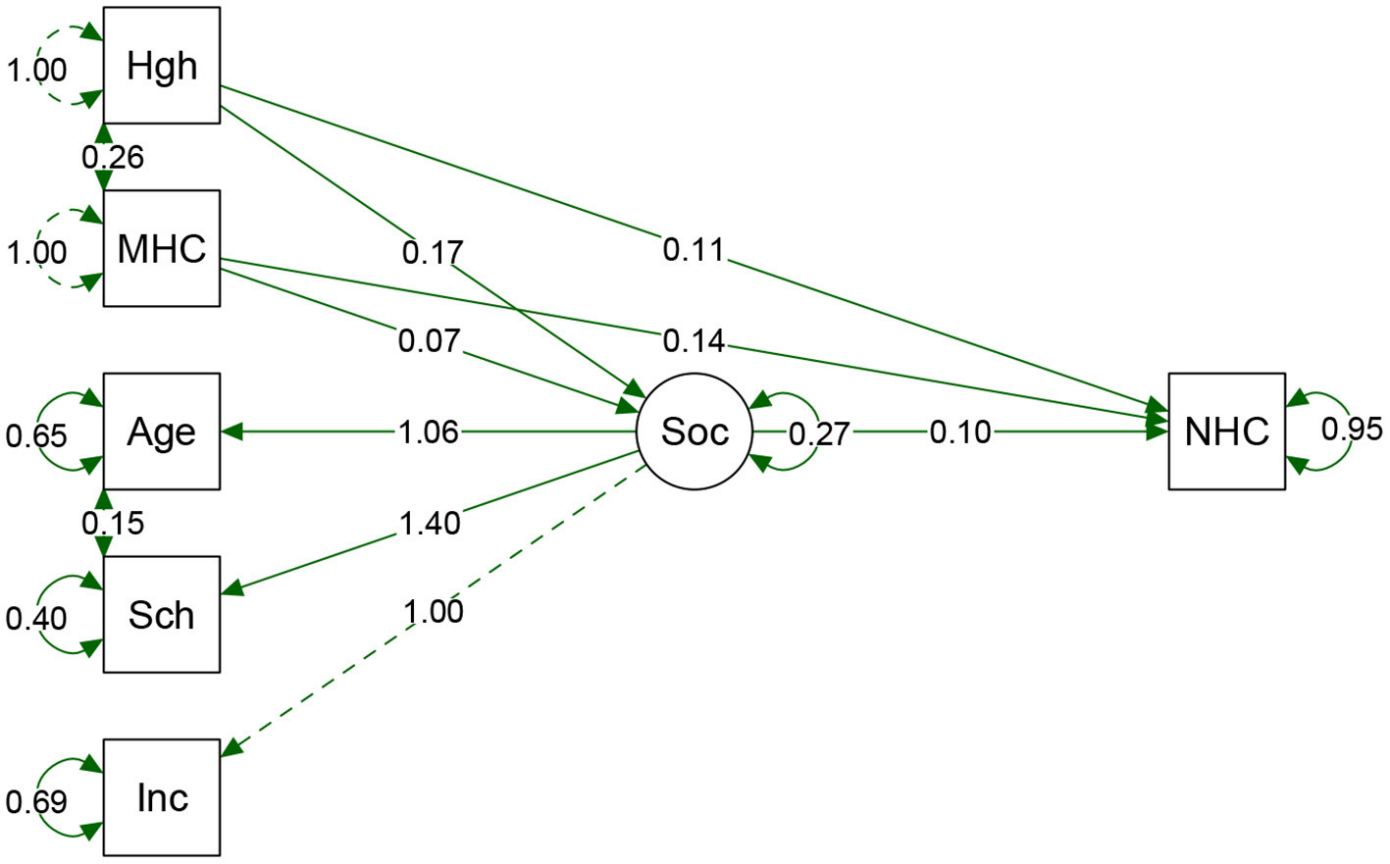
Result from path analyses of the total sample. Hgh, height; MHC, Maternal HC; Sch, Schooling. Inc, Income; Soc, Socio (latent variable); NHC, Neonatal HC. Arrow direction indicates direct and indirect effects of variables that predict NHC. The direction of Socio arrows for the three predecessor variables shows the contribution of each indicator value. As far as we could verify; schooling had a greater weight on Socio variable. Circles show the standard error of a parameter. Model fits: *p*-value = <0.0001. Chi-square (X^2^) = 0.340, Degree of Freedom (df) = 5. X^2^/df = 0.068. CFI = 0.999, TLI = 0.997. RMSEA = 0.013 (95%CI 0.000–0.052). Adjustment parameters suggested that the model is acceptable, indicating that the composition of these variables could explain the effects on the proportion of NHC.

The first model was constructed by using maternal anthropometric variables influenced by socioeconomic variables as a predictor path for neonatal HC outcome. Social variables were selected to avoid correlation and multicollinearity effects, such as color and region. Socioeconomic status was defined by three indicators: income, schooling, and age. For this purpose, we developed a latent variable that associated socioeconomic characteristics, named “Socio” (Figure 3). Adjusting the ratio reduces the confounding effects of the environment, which in this case was "Socio.” The significance of direct, indirect, and total effects was tested with path analysis for the total sample (Table 3).

**Table 3:** Regression parameters from “Path Analysis” using latent variables and defined parameters.

**Regression**	**Total**		
	**Estimate**	**z-value**	***p*** **-value**
NHC ~ MHC (A)	0.144	4.021	<0.001
NHC ~ Height (B)	0.106	2.758	0.006
NHC ~ Socio (C)	0.103	1.248	0.212
Socio ~ MHC	0.067	2.694	0.007
Socio ~ Height	0.172	5.343	<0.001
Effect			
TIE = A + B	0.250	0.047	<0.001
TE = TIE + C	0.026	0.019	0.179
Observations		812

*Associations between estimates of NHC among anthropometric parameters and latent variables. TIE, Total Indirect Effect; TE, Total Effect. A path coefficient indicates the direct effect of a variable assumed to be the cause in another variable assumed to be an effect. P-value estimates the significance of each effect on NHC size*.

*Values in bold mean they are significant*.

We repeated the process of the Path analysis method, searching for new explanatory factors per region. In the Northeast, all parameters were significant, including Socio as the latent variable (Table 4).

**Table 4:** Regression parameters from “Path Analysis” using latent variables and defined parameters according to regions.

**Regression**	**South/Southeast**			**Northeast**		
	**Estimate**	**z-value**	***p*** **-value**	**Estimate**	**z-value**	***p*** **-value**
NHC ~ MHC (A)	0.231	4.376	<0.001	0.098	2.074	0.038
NHC ~ Height (B)	0.058	1.034	0.301	0.159	3.267	0.001
NHC ~ Socio (C)	0.264	1.672	0.095	0.202	1.989	0.047
Socio ~ MHC	0.029	0.894	0.371	0.050	1.844	0.065
Socio ~ Height	0.114	2.373	0.018	0.117	2.919	0.004
Effect						
TIE = A + B	0.289	4.202	<0.001	0.256	4.211	<0.001
TE = TIE + C	0.076	1.749	0.080	0.052	2.028	0.043
Observations		357			455	

*Associations between estimates of NHC among anthropometric parameters and latent variables. SIE: Specific Indirect Effect. TIE, Total Indirect Effect; TE, Total Effect*.

*Northeast has a slightly significant p-value, and was acceptable to explain the observation that Socio and MHC difference were different from zero. A path coefficient indicates the direct effect of a variable assumed to be a cause in another variable assumed to be an effect. P-value estimates the significance of each effect on NHC size*.

*Values in bold mean they are significant*.

**Figure 4:**
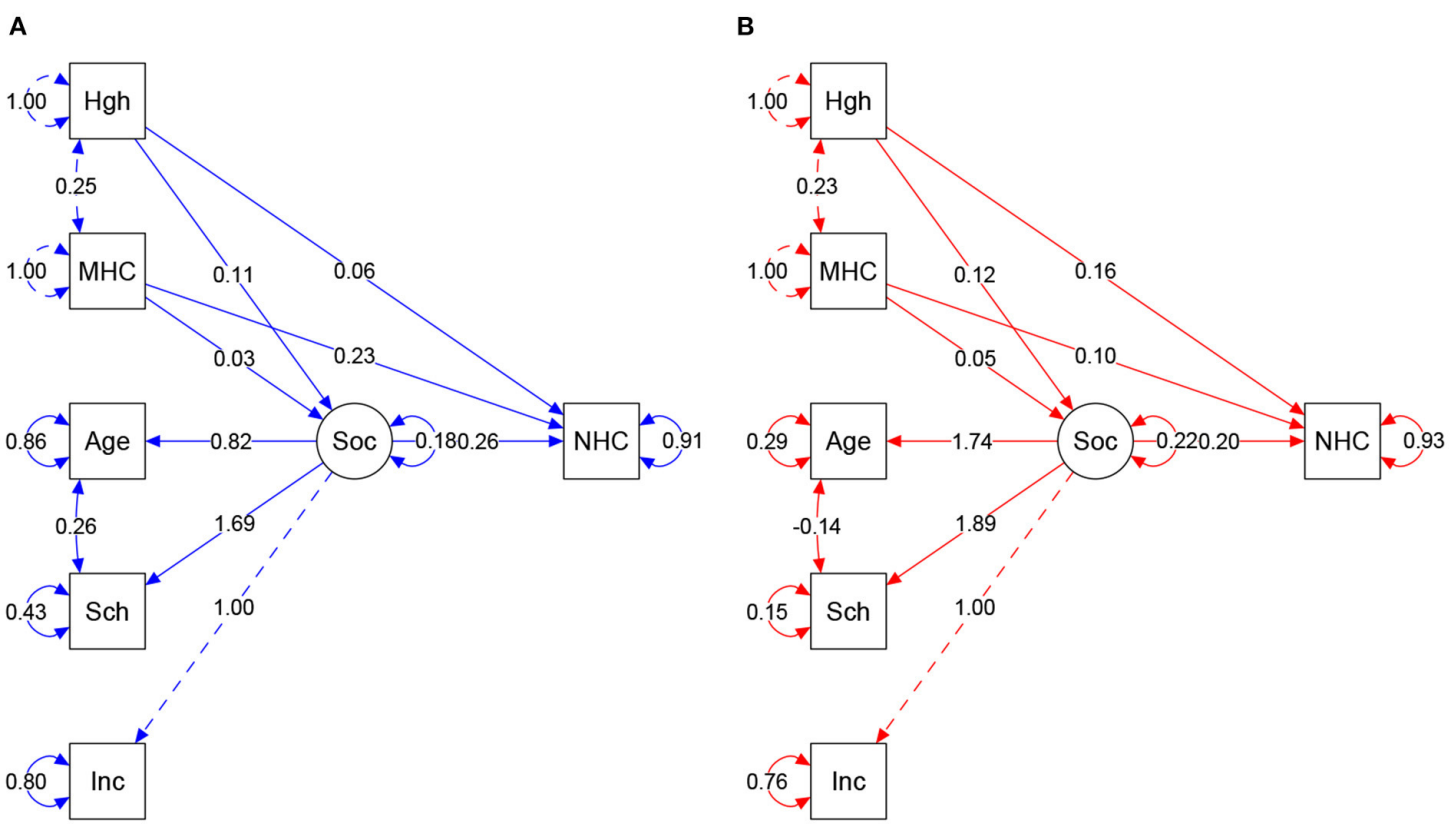
**(A,B)** Result of structural equation model according to regions. Colors = Blue: South/Southeast, Red: Northeast. Arrow direction indicates direct and indirect effects of variables in the prediction of NHC. Values show regression and covariances among variables. Circles show standardized parameter values. MHC, Maternal HC; Sch, Schooling; Inc, Income; Soc, Socio (latent variable); NHC, Neonate HC. Model fits: **South/Southeast**: *p*-value = <0.0001. Chi-square (X^2^) = 0.109, Degree of Freedom (df) = 5. X^2^/df = 0.0218. CFI = 0.981. TLI = 0.946. RMSEA = 0.047 (95%CI 0.000–0.096). **Northeast**: *p*-value = <0.0001. Chi-square (X^2^) = 0.130. df = 5. X^2^/df = 0.026. CFI = 0.991. TLI = 0.976. RMSEA = 0.039 (IC 95% 0.000–0.083). Plausibility indexes were considered capable of showing the influence of variable interaction, explaining the proportion of NHC.

Figure 4 shows the path and differences obtained in both regions and influence of maternal measures and social variables as predictive of neonatal HC.

## Discussion

This study reinforces the hypothesis that maternal head circumference has an influence on neonatal response. As a differential, we evaluated the effects of combined maternal measurements in association with social factors defined by a latent variable. In addition, we assessed these effects in populations which was previously accessed showing a significant difference among nutritional profile (20), as also their anthropometry and social conditions. Maternal and neonatal head circumference is transmitted to future generations, characterizing a transgenerational cycle of maternal nutrition and social inequality.

Nutritional indicators report food inadequacies supporting interventions and clinical decisions. The strength of more than one indicator can clarify the extent of nutrition conditions and their consequences (21). Low HC was indicated as a proxy to screening children on a neurodevelopment risk and was associated factors poverty and malnutrition in LMIC (22). Low HC was observed in maternal and child low-income populations and it was related to poverty and maternal food deprivation (23). Study with Indian children reinforces this finding by positively associating head circumference, developmental quotient and severe malnutrition (24). The malnutrition impacts extend beyond carrying on futures consequences as stunted children are constantly disadvantaged, have difficulty learning, and have less opportunities to support themselves and their families (25).

Malnutrition may be caused by food shortage, predominantly in low- and middle-income countries. In high-income countries, nutritional transition bears a double burden of obesity-related chronic diseases. The intergenerational effects on linear growth are not new, correlating with child stunting, even after adjusting for socioeconomic status. Over the span of a generation, it would be necessary to reverse damages due to maternal undernutrition, considering that little can be achieved in a single generation (26). As secondary effects of this cycle, a study showed a significant association between HC, malnutrition and a lower intellectual coefficient of the population (27). This relationship extended to maternal and paternal HC, and both were predictive of neonatal HC measurements until age two (23). The Global Nutrition Report 2020 has shed light on cycles of global malnutrition, alerting to the perpetuation of inequalities in countries, mainly affecting more socioeconomically disadvantaged populations (28).

The genetic potential can be influenced by epigenetic mechanisms in populations with different nutrition and environmental conditions. Epigenetic mechanisms can be considered a link between environmental stimuli and being able to influence the phenotype later in life (29). Global evidence of growth failure showed direct relation to low nutrient intake, disease burden, and intergenerational transfer. The authors propose actions before and during pregnancy such as improving better nutrition, education, and broad health care (6). Other analysis proposals expanded the causes that maintain stunting in populations and highlighted factors such as the influence of agricultural productivity, gender equality, women's education, water and sanitation infrastructure, access to health services and fertility rates being the targets of nutrition-sensitive interventions (30). Adult height is a bridge capable of reflecting the health of generations, being indicated as a useful tool to monitor maternal health conditions and offspring outcomes (31). We have analyzed it by associating it with mother and social factors to predict neonate HC. Using a structured equation model, our analysis indicated that head circumference measurements of infants born to women from the Northeast were more vulnerable to the effects of maternal nutrition and combined social conditions.

Other social inequalities are reported in the literature such as the effects of poverty, strongly associated with brain volume. Countries with socially vulnerable populations show lower gray matter volume in the temporal and frontal lobes and hippocampus. A decrease in brain volume is proportionally greater in regions that have a higher percentage of poverty-stricken individuals, and is directly related to income (32). A study that evaluated 3,383 Dutch children, reported the influence of socioeconomic differences in head circumference in early childhood. Children of mothers with low education had a smaller HC (33). Cognitive capacity depends on the development of a healthy brain, and this organ is the basis of our civilization. For the development of society, it is fundamental to invest in the promotion and protection of brain development in children (34). The influence of these structural brain alterations is associated with learning skills and school performance. Impairment is more commonly observed in children from low-income families. Lower brain development impacts negatively on academic performance in children. Recent advances were made in another study that described the extension of deleterious effects of poor maternal nutrition before conception. Linear growth, developmental epigenetics, and brain and neurocognitive development were analyzed, indicating that malnutrition in early life may be irreversible (35). In Brazil, there is still inequality between country regions. Women from the Northeast have lower income, schooling, and less paid work than women from the South/Southeast (20). Similarly, our study showed a significant effect of schooling in Northeastern women, where each year of lower education reduced neonatal HC by 0.31 cm A similar result was obtained by a study in women of low economic status, showing low maternal education associated with slower fetal growth with a greater effect on head growth compared to other parts of the body (36). Moreover, HC reflects brain size which is linked to cognitive function. The poor head circumference growth reflects the child's malnutrition, the vigilance can provide an understanding between poverty and cognitive development (37).

In the last decades, has been observed that in low- and middle-income countries, linear growth does not be recovered, even when corrected age is used. Children suffering from any type of nutritional deprivation had a lower growth rate according to the population evaluated, and failed to reach the growth velocity expected for ages 2 to 5 years (38). There is an urgency of attention to the high risk posing over all forms of malnutrition over mortality and morbidity that is still increased by the combined effect caused by unsafe sex, alcohol, drug and tobacco use (25).

Evaluation of children in low- and middle-income countries showed that malnutrition was prevalent in 50% of the children according to HC measurement. Furthermore, girls had a greater risk of malnutrition. Malnourished girls carry a biological burden resulting in a whole perpetual cycle. HC measurements should be followed to decrease the burden of malnutrition in children (39). The conventional definition of short height is related to poor nutrition and poor social condition, affecting children from low- and middle-income countries (LMIC) (40). However, an isolated analysis is unable to confirm that short stature is a proxy indicator of malnutrition (41). The influence of maternal inheritance on offspring development is termed “Intergenerational cycle of growth failure.” This relationship shows the impact of maternal nutrition in different countries. Of all the measurements taken, the authors concluded that HC varied the most, indicating that populations diverge in HC measurement, particularly according to nutritional status of children (42). Moreover, another approach showed an association between maternal malnutrition and its influence on the size and morphology of the placenta. As a result, the capacity to transport nutrients to the fetus is reduced, triggering an epigenetic effect that is perpetuated throughout postnatal life (43).

A limitation of this study was its inability to differentiate the nutritional contribution of the mother in the past. Also, it is important to proceed with future research, the evaluation of the inter-and intra-observed variability of the maternal and neonatal anthropometric measurements which was not carried out in this study. Nevertheless, this is an exploratory study with no intention of exhausting the subject. It alerts researchers to the topic in countries with a social abyss such as Brazil, and proposes a to make a further investigation of nutrition, along with its influence on social inequalities in different populations.

In conclusion, this study highlights the potential intergenerational influence of maternal nutrition. It also suggests that social status has an influence on maternal and neonatal HC measurements. Measurements are possibly affected by maternal nutrition and are more relevant in socially deprived families. The mechanism seems to be mediated by the effect of both HC measurements with root causes in different social conditions. Epigenetics probably plays a role in these mechanisms and warrants further studies. In women from different populations, attention to nutrition could activate/deactivate epigenetic mechanisms for inherited growth potential, and help break this adverse intergenerational cycle.

## Data availability statement

The raw data supporting the conclusions of this article will be made available by the authors, without undue reservation.

## Ethics statement

The studies involving human participants were reviewed and approved by the Institutional Review Boards of all participating centers (coordinating center protocol 20182318.8.0000.5404 from the University of Campinas), in addition to the National Ethics Committee for Research (CONEP). The patients/participants provided their written informed consent to participate in this study.

## Author contributions

MM, RS, RP, and JC designed the study. MM, RS, JM, IC, FF, DL, ER, KF, and JV conducted data collection. MM, JC, and MV conducted data analysis. MM wrote the first draft of the manuscript, reviewed initially by JC. All authors have access and participated in the interpretation of results, read, and agreed to the published version of the manuscript.

## Funding

This study was granted jointly by the Brazilian National Research Council (CNPq) (Award 401636/2013–5) and the Bill and Melinda Gates Foundation (Grant OPP1107597).

## Conflict of interest

The authors declare that the research was conducted in the absence of any commercial or financial relationships that could be construed as a potential conflict of interest.

## Publisher's Note

All claims expressed in this article are solely those of the authors and do not necessarily represent those of their affiliated organizations, or those of the publisher, the editors and the reviewers. Any product that may be evaluated in this article, or claim that may be made by its manufacturer, is not guaranteed or endorsed by the publisher.

## References

[1] Roseboom T de Rooij S Painter R. The Dutch famine and its long-term consequences for adult health. Early Hum Dev. (2006) 82:485–91. 10.1016/j.earlhumdev.2006.07.001 16876341

[2] Chen X Zhao D Mao X Xia Y Baker PN Zhang H. maternal dietary patterns and pregnancy outcome. Nutrients. (2016) 8:351. 10.3390/nu8060351 27338455 PMC4924192

[3] Stephenson J Heslehurst N Hall J Schoenaker DAJM Hutchinson J Cade JE. Before the beginning: nutrition and lifestyle in the preconception period and its importance for future health. Lancet. (2018) 391:1830–41. 10.1016/S0140-6736(18)30311-8 29673873 PMC6075697

[4] Colón-Ramos U Racette SB Ganiban J Nguyen TG Kocak M Carroll KN. Association between dietary patterns during pregnancy and birth size measures in a diverse population in Southern US. Nutr. (2015) 7:1318–32. 10.3390/nu7021318 25690420 PMC4344590

[5] Goldstein RF Abell SK Ranasinha S Misso ML Boyle JA Harrison CL. Gestational weight gain across continents and ethnicity: systematic review and meta-analysis of maternal and infant outcomes in more than one million women. BMC Med. (2018) 16:153. 10.1186/s12916-018-1128-1 30165842 PMC6117916

[6] Bhutta ZA Akseer N Keats EC Vaivada T Baker S Horton SE. How countries can reduce child stunting at scale: Lessons from exemplar countries. Am J Clin Nutr. (2020) 112:894S−904S. 10.1093/ajcn/nqaa153 32692800 PMC7487427

[7] Dupont C Castellanos-Ryan N Séguin JR Muckle G Simard MN Shapiro GD. The predictive value of head circumference growth during the first year of life on early child traits. Sci Rep. (2018) 8:1–9. 10.1038/s41598-018-28165-8 29959368 PMC6026134

[8] Leary S Fall C Osmond C Lovel H Campbell D Eriksson J. Geographical variation in relationships between parental body size and offspring phenotype at birth. Acta Obstet Gynecol Scand. (2006) 85:1066–79. 10.1080/00016340600697306 16929411 PMC2655054

[9] Rice F Thapar A. Estimating the relative contributions of maternal genetic, paternal genetic and intrauterine factors to offspring birth weight and head circumference. Early Hum Dev. (2010) 86:425–32. 10.1016/j.earlhumdev.2010.05.021 20646882 PMC2954294

[10] Cecatti JG Souza RT Sulek K Costa ML Kenny LC McCowan LM. Use of metabolomics for the identification and validation of clinical biomarkers for preterm birth: Preterm SAMBA. BMC Pregnancy Childbirth. (2016) 16:212. 10.1186/s12884-016-1006-9 27503110 PMC4977855

[11] Ministério da. Saude do Brasil. Orientações para a coleta e análise de dados antropométricos em serviços de saúde 1st ed, ed Secretaria de Atenção à Saúde Departamento de Atenção Básica Brasilia. (2011).

[12] Villar J Ismail LC Victora CG Ohuma EO Bertino E Altman DG. International standards for newborn weight, length, and head circumference by gestational age and sex: The Newborn Cross-Sectional Study of the INTERGROWTH-21st Project. Lancet. (2014) 384:857–68. 10.1016/S0140-6736(14)60932-6 25209487

[13] Brasil. Ministério da Saúde. Secretaria de Vigilância em Saúde. Departamento de Vigilância das Doenças Transmissíveis. Protocolo de vigilância e resposta à ocorrência de microcefalia e/ou alterações do sistema nervoso central (SNC). Brasília (2015).

[14] Noble M Wright G Dibben C Smith GAN McLennam D Anttila C. The English Indices of Deprivation 2004 (revised). in Office of the Deputy Prime Minister (ODPM). (2005).

[15] IBGE. Instituto Brasileiro de Geografia e Estatística Coordenação de População e Indicadores Sociais. Síntese de Indicadores Sociais. 1st ed. ed. IBGE Rio de Janeiro (2021).

[16] Joseph Hair F,; R.E. Willian Black C.; Barry Babin J.; Rolpf Anderson E.; Ronald TL. Modelagem de Equações Estruturais, in Análise Multivariada de Dados (Porto Alegre: Bookman), 539–586.

[17] Hu LT Bentler PM. Cutoff criteria for fit indexes in covariance structure analysis: Conventional criteria versus new alternatives. Struct Equ Model A Multidiscip J. (2009) 6:1–55. 10.1080/10705519909540118

[18] Lunde A Melve KK Gjessing HK Skjærven R Irgens LM. Genetic and environmental influences on birth weight, birth length, head circumference, and gestational age by use of population-based parent-offspring data. Am J Epidemiol. (2007) 165:734–41. 10.1093/aje/kwk107 17311798

[19] R Core Team. R: A Language and Environment for Statistical Computing. R Found Stat Comput. (2020) Available online at: https://www.r-project.org (accessed July 4, 2022).

[20] Miele MJ Souza RT Calderon IM Feitosa FE Leite DF. Profile of Different Food Choices, Habits and The Nutritional Density of Food in Nulliparous Women From Different Regions of Brazil. (2020).

[21] Habicht JP Pelletier DL. The importance of context in choosing nutritional indicators. J Nutr. (1990) 120:1519–24. 10.1093/jn/120.suppl_11.1519 2243298

[22] Connery A Colbert A Lamb M. Head circumference may be the best proxy for neurodevelopmental risk in children in low-resource settings. Arch Dis Child. (2022) 12: 323216. 10.1136/archdischild-2021-323216 35074831

[23] Sindhu KN Ramamurthy P Ramanujam K Henry A Bondu JD John SM. Low head circumference during early childhood and its predictors in a semi-urban settlement of Vellore, Southern India. BMC Pediatr. (2019) 19:1–11. 10.1186/s12887-019-1553-0 31170939 PMC6552319

[24] Tiwari K Goyal S Malvia S. Impact of malnutrition on head size and development quotient. Int J Res Med Sci. (2017) 5:3003–6. 10.18203/2320-6012.ijrms2017297711515234

[25] Global Panel on Agriculture and Food Systems for Nutrition. Food systems and diets: Facing the challenges of the 21st century. London, UK (2016).

[26] Martorell R Zongrone A. Intergenerational influences on child growth and undernutrition. Paediatr Perinat Epidemiol. (2012) 26:302–14. 10.1111/j.1365-3016.2012.01298.x 22742617

[27] Ivanovic DM Leiva BP Pérez HT Olivares MG Díaz NS Urrutia MSC. Head size and intelligence, learning, nutritional status and brain development. Head, IQ, learning, nutrition and brain Neuropsychologia. (2004) 42:1118–31. 10.1016/j.neuropsychologia.2003.11.022 15093150

[28] GNR. 2020 Global Nutrition Report. Glob Nutr Rep. (2020) Availableonline at: https://globalnutritionreport.org/reports/2020-global-nutrition-report/ (accessed July 4, 2022).

[29] Tozzi MG Moscuzza F Michelucci A Scaramuzzo RT Cosini C Chesi F. Nutrition, epigenetic markers and growth in preterm infants. BMC. (2019) 34:3963–8. 10.1080/14767058.2019.1702952 31842645

[30] Guo G Stearns E. The social influences on the realization of genetic potential for intellectual development. Soc Forces. (2002) 80:881–910. 10.1353/sof.2002.0007

[31] Perkins JM Subramanian S V Smith GD Özaltin E. Adult height, nutrition, and population health. Nutr Rev. (2016) 74:149–65. 10.1093/nutrit/nuv105 26928678 PMC4892290

[32] Blair C Raver CC. Poverty, stress, and brain development: new directions for prevention and intervention. Acad Pediatr. (2016) 16:S30–6. 10.1016/j.acap.2016.01.010 27044699 PMC5765853

[33] Bouthoorn SH van Lenthe FJ Hokken-Koelega ACS Moll HA Tiemeier H Hofman A. Head circumference of infants born to mothers with different educational levels; the Generation R Study. PLoS One. (2012) 7:e39798. 10.1371/journal.pone.0039798 22768125 PMC3387269

[34] Luby JL. Povertys most insidious damage: the developing brain. JAMA Pediatr. (2015) 169:810–1. 10.1001/jamapediatrics.2015.1682 26191940

[35] Leroy JL Frongillo EA Dewan P Black MM Waterland RA. Can children catch up from the consequences of undernourishment? evidence from child linear growth, developmental epigenetics, and brain and neurocognitive development. Adv Nutr. (2020) 11:1032–41. 10.1093/advances/nmaa020 32584399 PMC7360439

[36] Silva LM Jansen PW Steegers EAP Jaddoe VWV Arends LR Tiemeier H. Mother's educational level and fetal growth: the genesis of health inequalities. Int J Epidemiol. (2010) 39:1250–61. 10.1093/ije/dyq069 20478844

[37] Miller LC Joshi N Lohani M Singh R Bhatta N Rogers B. Head growth of undernourished children in rural Nepal: association with demographics, health and diet. Paediatr Int Child Health. (2016) 36:91–101. 10.1080/20469047.2015.1133517 27077633

[38] Leroy JL Ruel M Habicht JP Frongillo EA. Using height-for-age differences (HAD) instead of height-for-age z-scores (HAZ) for the meaningful measurement of population-level catch-up in linear growth in children less than 5 years of age. BMC Pediatr. (2015) 15:9. 10.1186/s12887-015-0458-9 26444012 PMC4595313

[39] Nitish M Sen J. Head circumference as an indicator of undernutrition among tribal pre-school children aged 2-5 years of North Bengal, India. Hum Biol Rev. (2016) 25:63–72. 10.4314/ejhs.v25i1.9 25733786 PMC4337084

[40] Black RE Victora CG Walker SP Bhutta ZA Christian P De Onis M. Maternal and child undernutrition and overweight in low-income and middle-income countries. Lancet. (2013) 382:427–51. 10.1016/S0140-6736(13)60937-X 23746772

[41] Scheffler C Hermanussen M Bogin B Liana DS Taolin F Cempaka PMVP. Stunting is not a synonym of malnutrition. Eur J Clin Nutr. (2019) 74:377–386. 10.1038/s41430-019-0439-4 31142828

[42] Natale V Rajagopalan A. Worldwide variation in human growth and the World Health Organization growth standards: a systematic review. BMJ Open. (2014) 4:735. 10.1136/bmjopen-2013-003735 24401723 PMC3902406

[43] Belkacemi L Nelson DM Desai M Ross MG. Maternal undernutrition influences placental-fetal development. Biol Reprod. (2010) 83:325–31. 10.1095/biolreprod.110.084517 20445129

